# P3a amplitude is related to conclusion specificity during category-based induction

**DOI:** 10.1371/journal.pone.0229515

**Published:** 2020-03-04

**Authors:** Hong Wang, Ruifang Cui, Changquan Long

**Affiliations:** 1 Key Laboratory of Cognition and Personality of MOE, Southwest University, Chongqing, China; 2 Center for Teaching and Learning Development, Chongqing Normal University, Chongqing, China; 3 The Clinical Hospital of Chengdu Brain Science Institute, MOE Key Lab for Neuroinformation, University of Electronic Science and Technology of China, Chengdu, China; 4 School of Life Science and Technology, Center for Information in Medicine, University of Electronic Science and Technology of China, Chengdu, China; Xuanwu Hospital, Capital Medical Universty, CHINA

## Abstract

Category-based induction involves the generalization of a novel property (conclusion property) to a new category (conclusion category), based on the knowledge that a category exemplar (premise category) has the respective novel property. Previous studies have shown that conclusion specificity (i.e., specific [S] or generic categories [G]) influences category-based induction. However, the timing of brain activity underlying this effect is not well known, especially with controlling the similarities of premise and conclusion categories between S and G arguments. In this study, the event-related potential (ERP) responses to category-based induction between S and G arguments were compared under both congruent (+, premise and conclusion categories are related) and incongruent (-, premise and conclusion categories are unrelated) arguments; additionally, the similarities of premise and conclusion categories between S and G arguments were controlled. The results showed that replicating this effect, S+ arguments have increased “strong” response rates compared to G+ arguments, suggesting that category-based induction is contingent on factors beyond matched similarities. Moreover, S arguments have more liberal inductive decision thresholds than G arguments, which suggest that conclusion specificity affects the inductive decision reflected by inductive decision thresholds. Furthermore, G+ arguments elicit greater P3a amplitudes than S+ arguments, which suggest greater attention resources allocation to the review of decisions for G+ arguments than that for S+ arguments. Taken together, the conclusion specificity effect during semantic category-based induction can be revealed by “strong” response rates, inductive decision thresholds, and P3a component after controlling the premise-conclusion similarity, providing evidence that category-based induction rely on more than simple similarity judgment and conclusion specificity would affect category-based induction.

## Introduction

Category-based induction, which is essential to learning and using knowledge [1], involves the use of knowledge about categorical relationships to generalize novel properties from category exemplars [2]. For example, learning that a hawthorn (premise category) contains molecular structure E5 (premise property) might lead one to infer that a fruit (conclusion category) also contains molecular structure E5 (conclusion property). This process has been variously termed as “categorical induction,” “property induction,” “feature induction,” and “induction projection” [3].

Previous studies have shown that specific (e.g., hawthorn) and generic (e.g., fruit) conclusion categories influence category-based induction; this phenomenon has been named “the conclusion specificity effect” [4]. According to the rational norms and rules of probability theory, the probability of drawing a “strong” conclusion decreases as generality increases [5, 6]. More specifically, a generalization that is made from a specific premise category to a more general conclusion category (G argument, e.g., inferring from “hawthorn has molecular structure E5” that “fruit has molecular structure E5”) is weaker than that which is made to a specific conclusion category, especially when the generic conclusion category includes the specific conclusion category (S argument, e.g., inferring from “hawthorn has molecular structure E5” that “jujube has molecular structure E5”). This is because the wider the range the conclusion category has, the larger the risk is that an erroneous generalization will be made.

However, the timing of brain activity underlying the effect of conclusion specificity on inductive decision during category-based induction is unclear. Event-related potentials (ERPs) can provide real-time information about cognitive processes with superior temporal resolution [7]. Previous ERP studies on category-based induction have used specific [8–10] and generic conclusions [11–13]. However, these studies ignored the influence of conclusion specificity. Indeed, Lei et al. (2017, 2019) measured ERP responses to inductive reasoning that were based on G and S arguments, wherein they focused on the influence of the hierarchical levels of premise categories [14] and the hierarchical distances between the premises and conclusions (e.g., inferences made from superordinate-to-basic-level categories and from basic-to-superordinate-level categories) [15]. However, Lei et al. (2017, 2019) neither directly compared the ERP responses of G and S arguments under standardized arguments, nor controlled the similarities between premise and conclusion categories.

The present study, therefore, aimed to explore the timing of brain activity underpinning the effect of conclusion specificity on inductive decision during category-based induction. This was done by comparing the ERP responses to inductive decisions made between G and S arguments, while the similarities of premises and conclusions between G and S arguments were matched. G arguments consisted of superordinate-level categories as conclusion categories; however, S arguments consisted of basic-level categories as conclusion categories. Furthermore, both congruent conclusions (+, i.e., the premise and conclusion belong to the same category) and incongruent conclusions (-, i.e., the premise and conclusion do not belong to the same category) were included. The conclusion categories and properties were presented separately to negate the influence of repetition [16, 17], categorical hierarchies [18], and the concreteness effects [19, 20] on conclusion categories. After the start of each experiment, participants were required to either respond with a response of either “strong” or “weak,” which is similar to Liang et al. (2016) [21]. When the conclusion properties were presented, ERP responses were measured and analyzed.

For behavior results, we predicted that S+ arguments would produce more responses of “strong” than G+ arguments, based on the effect of conclusion specificity on inductive decision. Moreover, the exemplar-based linear ballistic accumulator model (the ex-LBA model) [2] suggested that a more “strong” inductive decision may be influenced by a lower inductive decision threshold (i.e., the amount of evidence required) and/or a more rapid speed of evidence accumulation (which is deemed as the similarities between the premises and conclusions). In accordance with the ex-LBA model [2], we hypothesized that S arguments may have a lower inductive decision threshold than G arguments. Meanwhile, we inferred that S and G arguments would have basically the same reaction times. As the similarities of premises and conclusions were matched between S and G arguments, the speed of evidence accumulation should be similar between S and G arguments based on the ex-LBA model [2]. If the inductive decision thresholds have no influence on reaction times, G and S arguments would have similar reaction times. Otherwise, S arguments would have shorter reaction times than G arguments.

For ERP results, we hypothesized that G+ arguments would elicit greater P3a amplitudes than S+ arguments. P3a component is a positive deflection that appears with a fronto-central brain distribution for a timespan of approximately 300–500 ms [22, 23]. Long et al. (2015) suggested that the P3a-like component is related to attention allocation to response review during category-based induction [8]. Therefore, we hypothesized that G+ arguments may necessitate greater attention allocation to supervise decisions because of an increased inductive conclusion range compared to S+ arguments, which in turn would elicit greater P3a amplitudes.

## Methods

### Ethical statements

This study was approved by the ethics review board of the Faculty of Psychology, Southwest University, Chongqing, China. Written informed consent was obtained from all participants. All procedures involved were in accordance with the seventh revision of the Declaration of Helsinki [24].

### Participants

Twenty-four healthy undergraduates (i.e., 16 females and 8 males) between the ages of 18 and 25 years (*M* = 21.52, *SD* = 1.93) participated in the ERP experiment. All participants were right-handed and had a normal or corrected-to-normal vision. A total of 25 undergraduate students (i.e., 17 females and 8 males), between the ages of 18 and 25 years (*M* = 21.31, *SD* = 2.81), evaluated the degree of similarity between the premise and conclusion for each trial. Participants who evaluated the degree of similarities were not included in the ERP experiment.

### Experimental design

The experiment had repeated measures factorial design. In this study, premise categories consist of basic-level categories. The novel properties (i.e., a series of molecular structures) were used as blank properties, in order to reduce the influence of background knowledge [25]. These molecular structures were represented by a capital letter and an Arabic numeral (e.g., X1, E5). An object from a premise category (e.g. hawthorn) was presented alongside a premise property (e.g., molecular structure X1), indicating that the premise property described the premise category (e.g., “hawthorn X1” indicated “hawthorn has molecular structure X1”).

Conclusion categories included specific (S) and generic (G) categories; whereas the former was a basic-level category (e.g., jujube), the latter was a superordinate-level category (e.g., fruit). In addition, to exclude participants from pressing only one fixed key without thinking carefully during the experiment (i.e., “strong” key), the conclusion categories could also be separated into two types: congruent (+) or incongruent (−) conclusions. For congruent conclusions, the premise and conclusion belong to the same category; however, in incongruent conclusions, the premise and conclusion do not belong to the same category. In each trial, conclusion category and conclusion property were presented separately. The reason for selecting this procedure was to negate the influence of repetition [16, 17], categorical hierarchies [18], and the concreteness effect [19, 20] on conclusion categories in G arguments.

### Materials

In the present study, the premise categories consisted of five generic categories, as follows: 18 types of vegetables, 6 types of fruits, 6 types of birds, 3 types of mammals, and 7 types of insects. For S+ arguments, 40 specific-level words, which comprised 18 types of vegetables, 6 types of fruits, 6 types of birds, 3 types of mammals, and 7 types of insects, were used for the conclusion categories. For S− arguments, 40 specific-level words, which comprised 18 types of clothing, 6 types of weapons, 6 types of tools, 3 types of furniture, and 7 types of electrical appliances, were used for the conclusion categories. G+ arguments consisted of five generic-level words, namely, vegetables, fruits, birds, mammals, and insects; whereas G− arguments contained another set of five generic-level categorical words, namely, clothing, furniture, weapons, electrical appliances, and tools. A total of 160 trials were conducted in the formal ERP experiment (40 trials per kind of arguments). Sixteen additional trials (4 trials per kind of arguments) were used for training purposes and were not replicated in the formal experiment.

Before the formal experiment, we matched the degree of the premise-conclusion categories similarity between the S and G arguments. Specifically, we created pairs under S arguments in which both the premise and conclusion categories were specific categories (e.g., hawthorn-jujube), and created pairs under G arguments in which the premise categories were the same to the S arguments while the conclusion categories were generic categories (e.g., hawthorn-fruit). Participants were required to decide the degree of similarity between the premise and conclusion categories on a 5-point Likert-type rating scale (i.e., 1 = least similar; 5 = most similar) for each premise-conclusion pair with a random sequence. For example, participants were given instructions such as, “please judge the degree of similarity between hawthorn and fruit on a 5-point scale; 1 represents the least similar and 5 represents the most similar.” Thus, total 160 pairs were required to make similarity judgment, each kind of arguments (S+, S-, G+, and G-) involved 40 pairs. After that, under congruent condition, we execute a pair-*t* test to compare the degree of the premise-conclusion similarity between S and G argument if the S and G argument share the same premise (e.g., executing a pair test to compare the degree of the premise-conclusion similarity between hawthorn-jujube argument and hawthorn-fruit argument). Under incongruent condition, we execute the same procedure to that under congruent condition. Finally, for each S-G argument pair, our results suggested that the degree of the premise-conclusion similarity between S and G arguments were similar (all *p* > 0.05). The statistical details of the similarity comparison for each S-G argument pair used in the formal experiment were showed in S1 Table.

### Procedures

**Fig 1:**
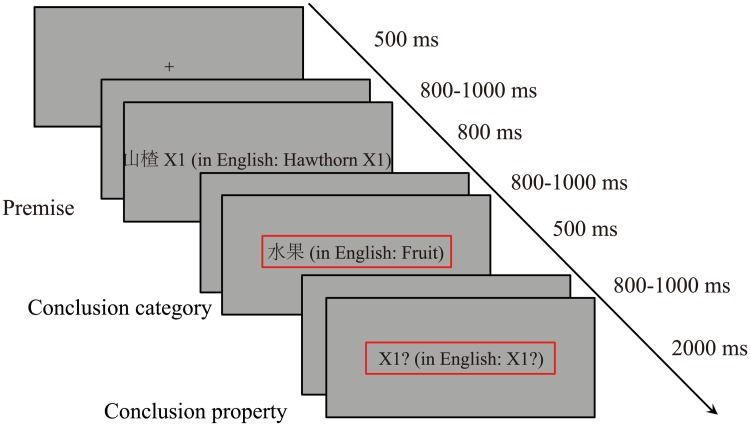
The experimental procedure.

Participants were seated 80 cm in front of a monitor. As shown in Fig 1, in each trial, a “+” sign was presented at the center of the computer screen; following this, a premise was presented (e.g., “hawthorn X1”). After the presentation of the premise, a conclusion category was presented (e.g., “fruit”). Next, a conclusion property was presented along with a question mark (e.g., “X1?”), which served as a cue for participants to provide a response (Fig 1). Participants were required to base their responses on the premise and to decide whether the respective conclusion was “strong” or “weak.” They were instructed to press either “1” (“strong”) or “2” (“weak”) with their right index finger or middle finger. The presentation order of trials is completely random, and participants were allowed to rest for 60 s for executing every 40 trials.

### EEG data acquisition and pre-processing

Electroencephalogram (EEG) readings from 64 electrode sites across the scalp were recorded using a Neuroscan cap (Neuroscan, Herndon, VA) with Ag/AgCl electrodes, while participants responded to the conclusion properties that were presented to them. Electrodes were positioned according to the International 10–20 system. A vertical electrooculogram (EOG) was recorded supraorbitally and infraorbitally from the left eye, and the horizontal EOG was recorded from the left versus the right orbital rim. The EEG and EOG were amplified using a SynAmps2 amplifier (Neuroscan) and digitized at a 500-Hz sample rate. The EEG and EOG were amplified with a bandpass filter of 0.05 to 200 Hz in alternating current (AC) mode. All interelectrode impedances were maintained below 5 kΩ.

Offline analyses were conducted in MATLAB 2014b (MathWorks, Natick, MA) using EEGLAB [26] and ERPLAB toolboxes [27]. EEG data were filtered using second-order IIR-Butterworth filters, with half-power cutoffs of 0.10 Hz (roll-off = 12 dB/oct) and 30 Hz (roll-off = 12 dB/oct) as high-pass [7] and low-pass filters, respectively. Subsequently, independent component analysis (ICA) was performed to correct components that are associated with eye movements and eye blinks. Next, ICA-corrected EEG data were re-referenced to the average of the left and right mastoids [7].

The epochs were segmented and time-locked to the conclusion properties stimulus onset. Because of lacking enough trials for the “weak” responses to congruent conclusions and for the “strong” responses to incongruent conclusions, we selected the “strong” responses to congruent conclusions and the “weak” responses to incongruent conclusions to be overlapped and averaged, which is similar to previous studies [11, 13]. Each epoch was 1,000 ms and included a 200 ms pre-stimulus baseline correction. Trials were excluded as noise by using a moving window peak-to-peak amplitude method [7], with a moving window width of 200 ms, a window step of 100 ms, and a threshold of 65 μV. The mean numbers of trials for each kind of arguments were as follows: 30.79 (*SD* = 5.45) for S+, 28.17 (*SD* = 5.33) for G+, 32.88 (*SD* = 4.56) for S−, and 32.96 (*SD* = 4.18) for G−.

### Data analyses

For behavioral data, two separate two-factor repeated measures analysis of variance (ANOVA) were conducted to analyze the “strong” response rates and reaction times for “strong” responses under congruent arguments and “weak” responses under incongruent arguments. The “strong” response rates referred to the proportion of the number of “strong” responses in all responses under each kind of argument. In these analyses, conclusion specificity (S, G) and conclusion congruency (+, −) were within-subject factors. With regard to the inductive decision thresholds, paired sample *t*-tests were used for parameter *c*, which is based on signal detection theory (SDT) [28], and parameter *Br*, which is based on the two-high threshold model (2HTM) [29]. The larger the parameter *c*, the more conservative the inductive decision thresholds are; whereas, the smaller the parameter *Br*, the more conservative the inductive decision thresholds are. To calculate parameters *c* and *Br*, the “strong” responses in the congruent conclusions were defined as “hits,” while the “weak” responses in the incongruent conclusions were defined as “correct rejections.”

For ERP data, considering the visual observation of ERP waveforms, the mean amplitudes of P3a were measured within 250–450-ms time window, after the presentation of conclusion properties. To increase statistical strength and reduce false effects [30], the F3, F1, Fz, F2, F4, FC3, FC1, FCz, FC2, and FC4 electrodes were selected and collapsed by averaging their values as a measure of the anterior region. Similarly, the CP3, CP1, CPz, CP2, CP4, P3, P1, Pz, P2, and P4 electrodes were selected and collapsed by averaging their values as a measure of the posterior region. A three-factor repeated measures ANOVA was used to analyze mean differences in P3a amplitudes with conclusion specificity (S vs. G), conclusion congruency (+ vs. -), and region (anterior vs. posterior) as the independent variables. Prior to running the analyses, Mauchly’s sphericity test was performed to test the assumption of sphericity; with regard to cases for which sphericity was violated (i.e., *p* < 0.05), the Greenhouse-Geisser correction was used. For all analyses, the level of statistical significance was specified as 0.05.

## Results

**Fig 2:**
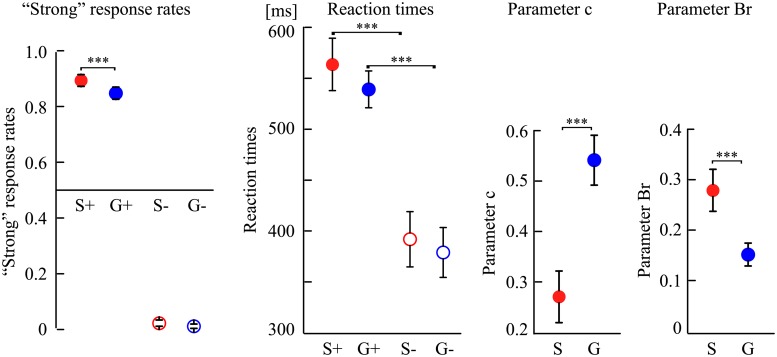
The “strong” response rates, reaction times, and inductive decision thresholds for each kind of arguments. S+ indicated arguments with specific congruent conclusion categories; G+ indicated arguments with generic congruent conclusion categories; S− indicated arguments with specific incongruent conclusion categories; and G− indicated arguments with generic incongruent conclusion categories. Error bars represent mean ± S.E.M. ***p < 0.001.

With respect to “strong” response rates, the interaction between conclusion specificity and conclusion congruency was significant (*F* [1, 23] = 7.69, *p* = 0.02, η_p_^2^ = 0.25). The main effect of both conclusion specificity (*F* [1, 23] = 22.99, *p* < 0.001, η_p_^2^ = 0.50) and conclusion congruency (*F* [1, 23] = 1015.17, *p* < 0.001, η_p_^2^ = 0.98) were also significant. The *post-hoc* analysis of conclusion specificity showed that the “strong” response rates were significantly higher in S+ arguments (*M* = 0.89, *SD* = 0.10) than in G+ arguments (*M* = 0.82, *SD* = 0.11, *F* [1, 23] = 16.82, *p* < 0.001, η_p_^2^ = 0.42). However, no significant difference in “weak” response rates emerged between the S− arguments (*M* = 0.03, *SD* = 0.05) and the G− arguments (*M* = 0.01, *SD* = 0.04, *F* (1, 23) = 3.18, *p* = 0.09, η_p_^2^ = 0.12). The *post-hoc* analysis of conclusion congruency showed that compared with the number of “strong” responses under congruent conclusions, incongruent conclusions had more “weak” responses for both G (*F* [1, 23] = 798.84, *p* < 0.001, η_p_^2^ = 0.97) and S arguments (*F* [1, 23] = 1037.51, *p* < 0.001, η_p_^2^ = 0.98) (Fig 2).

With regard to inductive decision thresholds, G arguments exhibited a more conservative inductive threshold than S arguments. The differences between S and G arguments on both parameters *c* (S: 0.27; G: 0.54) and *Br* (S: 0.29; G: 0.14) were significant (*c*: *t* [1, 23] = -5.10, *p* < 0.001, Cohen’ *d* = -1.08; *Br*: *t* [1, 23] = 4.21, *p* < 0.001, Cohen’ *d* = 0.87) (Fig 2).

With regard to reaction times, the interaction between conclusion specificity and conclusion congruency was not significant (*F* [1, 23] < 1, *p* = 0.47, η_p_^2^ = 0.02). The main effect of conclusion congruency was significant (*F* [1, 23] = 85.75, *p* < 0.001, η_p_^2^ = 0.79), thereby indicating that the reaction times were shorter in the incongruent conclusions than in the congruent conclusions. Another finding pertained to the main effect of conclusion specificity, which was not significant. This suggests that S and G arguments elicit similar reaction times (*F* [1, 23] = 3.92, *p* = 0.06, η_p_^2^ = 0.15) (Fig 2).

**Fig 3:**
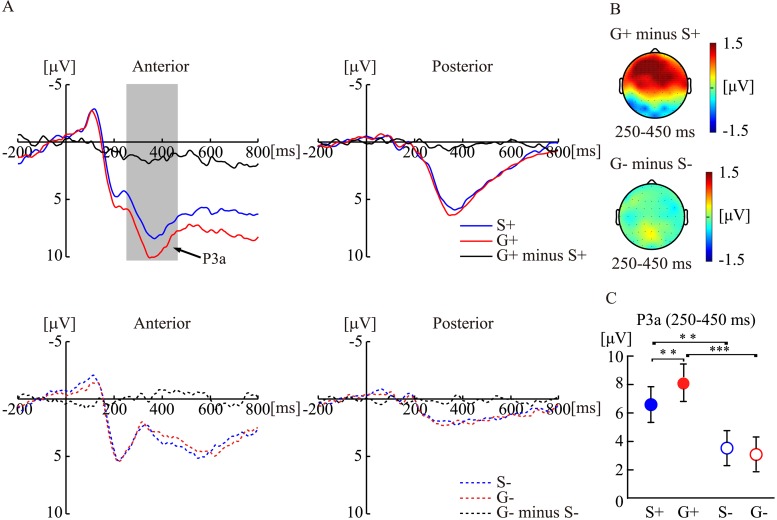
P3a amplitudes to the effect of conclusion specificity in the congruent and incongruent conclusions. A) The grand-averaged waveforms elicited by G+ and S+ arguments and the difference waveforms between G+ and S+ arguments (G+ minus S+) in the anterior and posterior regions; and the grand-averaged waveforms elicited by G− and S− arguments and the difference waveforms between G− and S− arguments (G− minus S−) in the anterior and posterior regions. B) The topographies of the difference waveforms at 250–450-ms time window (G+ minus S+); and the topographies of the difference waveforms at 250–450-ms time window (G− minus S−). C) P3a amplitudes for each kind of arguments. The anterior region indicates the mean amplitudes of F3, F1, Fz, F2, F4, FC3, FC1, FCz, FC2, and FC4 electrodes; the posterior region indicates the mean amplitudes of CP3, CP1, CPz, CP2, CP4, P3, P1, Pz, P2, and P4 electrodes. S+ indicated arguments with specific congruent conclusion categories; G+ indicated arguments with generic congruent conclusion categories; S− indicated arguments with specific incongruent conclusion categories, and G− indicated arguments with generic incongruent conclusion categories.

The results of analyses conducted with P3 amplitudes showed that the interactions among conclusion specificity, conclusion congruency, and region were significant (*F* [1, 23] = 8.19, *p* = 0.008, η_p_^2^ = 0.26). Therefore, we analyzed the interaction between conclusion specificity and conclusion congruency within each region, namely, the anterior and posterior regions. With regard to the anterior region, the results showed that the interaction between conclusion specificity and conclusion congruency was significant (*F* [1, 23] = 5.80, *p* = 0.02, η_p_^2^ = 0.20). The *post-hoc* analysis of conclusion specificity suggested that S+ arguments elicited significantly smaller P3 amplitudes (*F* [1, 23] = 9.47, *p* = 0.005, η_p_^2^ = 0.29) than G+ arguments; however, S− and G− arguments elicited similar amplitudes (*F* [1, 23] < 1, *p* = 0.53, η_p_^2^ = 0.02). Further, the *post-hoc* analysis of conclusion congruency showed that congruent conclusions elicited significantly greater amplitudes than incongruent conclusions. This finding was significant for both S (*F* [1, 23] = 9.60, *p* = 0.005, η_p_^2^ = 0.29) and G (*F* [1, 23] = 31.22, *p* < 0.001, η_p_^2^ = 0.57) arguments (Fig 3).

With regard to the posterior region, results indicated that there was no significant interaction (*F* [1, 23] < 1, *p* = 0.58, η_p_^2^ = 0.01) between conclusion specificity and conclusion congruency. Further, the main effect of conclusion specificity was not significant (*F* [1, 23] = 2.14, *p* = 0.16, η_p_^2^ = 0.01), whereas the main effect of conclusion congruency was significant (*F* [1, 23] = 49.58, *p* < 0.001, η_p_^2^ = 0.68). This suggests that congruent conclusions elicit greater P3 amplitudes than incongruent conclusions (Fig 3).

**Fig 4:**
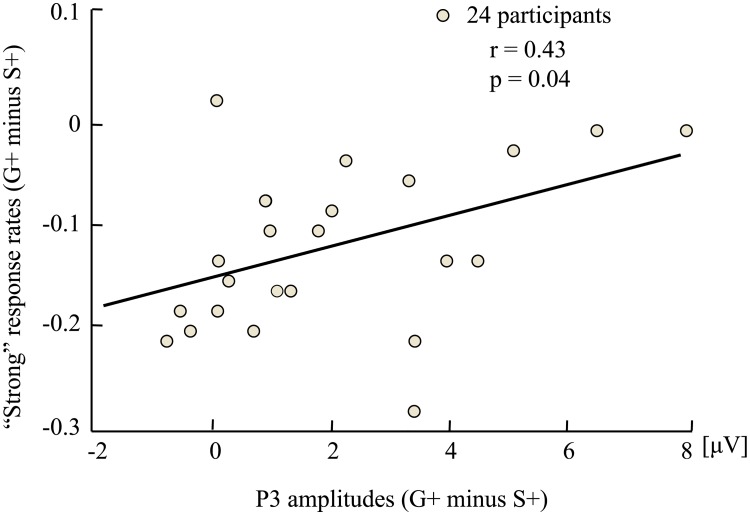
Pearson correlation analysis between the difference of P3a amplitudes (G+ minus S+) and the difference of “strong” response rates (G+ minus S+). S+ indicated arguments with specific congruent conclusion categories; G+ indicated arguments with generic congruent conclusion categories.

To explore the relationship between P3a amplitudes and behavioral results, we calculated Pearson correlation coefficient between the difference of P3a amplitudes of S+ and G+ arguments (G+ minus S+) and the differences of “strong” response rates of S+ and G+ arguments (G+ minus S+). The Pearson correlation coefficient between the difference of P3a amplitudes of S+ and G+ arguments (G+ minus S+) and the difference of inductive decision thresholds (G minus S) was also calculated. The results showed that the differences in P3a amplitudes (G+ minus S+) were positively correlated with the differences in “strong” response rates (G+ minus S+) (*r* = 0.43, *p* = 0.04, 95% CI [0.03, 0.71]) (Fig 4), whereas no other significant correlation was found (all *ps* > 0.05).

## Discussion

Previous ERP studies ignored the ERP responses to the effect of conclusion specificity on inductive decision during category-based induction after controlling for the similarity between premise and conclusion categories. To explore this issue, the present study compared inductive reasoning based on S and G arguments by using the ERP technique, matching the premise-conclusion categories’ similarities between S and G arguments. The results suggest that the effect of conclusion specificity on inductive decision during category-based induction is revealed by “strong” response rates (i.e., the proportion of the number of “strong” responses in all responses under each kind of argument), inductive decision thresholds, and P3a amplitudes. However, the effect of conclusion specificity on inductive decision during category-based induction is not revealed by reaction times.

As stated above, the effect of conclusion specificity on inductive decision during category-based induction is revealed by the “strong” response rates. In the present study, S+ arguments have more “strong” response rates than G+ arguments. This finding is in accordance with the propositions of probability theory [5, 6], which suggests that increasing the conclusion generality of G arguments reduces the probability of accepting inductive reasoning. Moreover, this fact suggests that category-based induction is contingent on factors beyond matched similarities, as the similarities between the premise and the conclusion of S and G arguments were matched in the present study. In addition, no significant differences in “strong” response rates emerged between S− and G− arguments. This can be explained by the fact that the conclusion categories were non-living beings in the incongruent conditions, which are unrelated to the premise categories. As a result, the similar lower rate of “weak” responses to conclusions were found in both S− and G− arguments.

The effect of conclusion specificity on inductive decision during category-based induction is also revealed by inductive decision thresholds. The ex-LBA model [2] hypothesized that the judgment of category-based induction may be influenced by inductive decision thresholds. However, few studies on category-based induction have measured inductive decision thresholds to test this hypothesis. One such study by Cui et al. (2018) showed that the inductive decision thresholds affect premise monotonicity during semantic category-based induction [11], which is based on SDT [28]. In the present experiment, S arguments exhibit a more liberal inductive decision threshold than G arguments based on both the SDT [28], and the 2HTM [29], providing additional evidence to support this hypothesis.

The effect of conclusion specificity on inductive decision during category-based induction is further supported by the P3a amplitudes. In the present study, G+ arguments elicited greater positive amplitudes than S+ arguments in a 250–450-ms time window with anterior scalp distribution. This positive deflection shows a similar time window and scalp distribution of P3a amplitudes; therefore, we infer that the differences between the ERP responses of S and G arguments are indeed the effects of P3a amplitudes.

In the literature, most studies demonstrated that P3a is related to attention processing. Some suggested that P3a is stimulus-driven, reflecting involuntary, exogenous attention to rare and unexpected stimuli [22, 31–36]. On the other hand, some researchers proposed that P3a is associated with top-down attention, as associated with goal oriented and task switch [37–39].

In the present study, we hypothesized that P3a is related to attention allocation for response review or response monitoring. Long et al. (2015) found properties violations elicit increased P3a-like amplitudes in a semantic category-based induction, suggesting that P3a-like is related to attention allocation for response review during category-based induction [8]. Folstein and Van Petten (2011) also suggested that the P3a amplitude-like component is associated with the recruitment of executive functions dependent on the prefrontal cortex [40]. Other studies also suggested that P3a is related to attention allocation for response inhibition process [41–45]. These convergent findings suggest that P3a or P3a-like amplitudes are associated with allocating attention to decision review. In the present study, G arguments indicate more conservative inductive decision thresholds than S arguments do. Therefore, when reviewing G+ arguments, participants may have allocated greater attention resources to the review of inductive decisions than when reviewing S+ arguments. As a result, P3a amplitudes may have been greater for G+ arguments than for S+ arguments.

Another potential explanation of P3a component in the present study is that P3a is related to beliefs updating. Several studies found that P3a amplitudes were varied during Bayesian inference and learning, indicating the changes of beliefs about hidden states given current observations, with a positive relationship between belief updating size and P3 amplitude [46–49]. In the present study, G+ have lower “strong” response rates than S+, suggested that G+ required larger belief updating size to accept the conclusions. Therefore, G+ elicited larger P3a amplitudes than S+.

However, the present study has some limitations. The first limitation is that the conclusion specificity effect on inductive decision during category-based induction is not implied by the participant’s reaction times. The ex-LBA model [2] suggests that reaction times to inductive reasoning may be influenced by the speed of evidence accumulation and/or inductive decision thresholds. In the present experiment, the speed of evidence accumulation was found to be similar for both S and G arguments, based on participants’ reaction times. As per the ex-LBA model, a possible explanation for this finding is that the speed of evidence accumulation is similar for premises and conclusions because the similarities between the S and G arguments were controlled. In contrast, as previously noted, the inductive decision thresholds are different between S and G arguments. The absence of a significant difference in the reaction times between S and G arguments, therefore, suggests that the effect of conclusion specificity on inductive decision during category-based induction is not reflected by the reaction times and inductive decision thresholds have no significant effect on reaction times. However, it doesn’t rule out the possibility that the results on reaction times may be caused by the special experimental procedure of the present study. Because we present the conclusion category and property separately with a 1000-ms blank screen. The speed difference between S and G may be reflected in the time window before the conclusion property is presented. Therefore, future study need test this result by a procedure that presents the conclusion category and property synchronously.

The second limitation is that we do not control the typicality of the premise categories. Previous studies suggested that the typicality of the premise categories affect inductive decision [9, 25]. However, in the present study, the premise categories were the same under S and G arguments, the typicality of the premise categories would have the same influence on both S and G argument. Moreover, the significant Pearson correlation between the difference of P3a amplitudes (G+ minus S+) and the differences of “strong” response rates (G+ minus S+) were found, which suggested that the potential confusion factors are well controlled in the present experiment. Nevertheless, further studies can measure the conclusion specificity effect during category-based induction after controlling the typicality of the premise categories.

The third limitation is that non-living beings were used as conclusion categories under incongruent conditions while living being were used as conclusion categories under congruent conditions. Previous studies suggested that there was significant difference of inductive decisions between living beings and non-living beings [13, 50]. Future studies require the consideration of incongruent conclusion categories using living being categories but different from its premise.

Finally, only single-premise category-based induction tasks were used in the present study, without comparing category-based induction between S and G arguments across multiple-premise tasks. Feeney (2007) found that participants tended to be more sensitive to the amount (premise monotonicity effect) and diversity (premise diversity effect) of the evidence for G arguments rather than S arguments during category-based induction. Thus, further research is required to explore the ERP responses to the effect of conclusion specificity during category-based induction using multiple-premise category-based induction tasks.

## Conclusions and implications

In the present study, category-based induction between S and G arguments was compared, and the similarities between premises and conclusions were controlled. The results showed that the effect of conclusion specificity on inductive decision during category-based induction is revealed by “strong” response rates, inductive decision thresholds, and P3a amplitudes. Specifically, S+ arguments have increased “strong” response rates compared to G+ arguments, which replicates the effect of conclusion specificity on inductive decision and suggests that category-based induction is contingent on factors beyond matched similarities. Moreover, S arguments have more liberal inductive decision thresholds than G arguments, which supports the hypothesis that conclusion specificity affects the inductive decision by inductive decision thresholds. Furthermore, S+ arguments elicit smaller P3a amplitudes than G+ arguments, which suggests that greater attention resources may be allocated to the review of decisions for G+ arguments than for S+ arguments. Taken together, our findings reveal that the conclusion specificity affect inductive decision during category-based induction after controlling the premise-conclusion similarity, showing by “strong” response rates, inductive decision thresholds, and P3a component which is related to attention resources allocation. The present study provided further evidence that category-based induction can go beyond simple similarity judgment, and has certain guiding significance for the material selection of category-based inductive tasks.

## Supporting information

S1 Table The degree of the premise-conclusion categories similarities that S and G arguments entailed for each trial in the present study. (DOCX)
