# Supplementary material for: P3a amplitude is related to conclusion specificity during category-based induction
Source: PLoS One. 2020 Mar 4;15(3):e0229515. doi: 10.1371/journal.pone.0229515 (PMC7055884; doi:10.1371/journal.pone.0229515)
Supplement: S1 Table — (DOCX) [file pone.0229515.s001.docx]

S1 Table. The degree of similarities between the premise and conclusion categories that S and G arguments entailed for each trial in the present study.

| **Premise category** | | **General category** | | ***M*** | ***SD*** | **Premise category** | | **Specific category** | | ***M*** | ***SD*** | ***F*** | ***p*** |
| --- | --- | --- | --- | --- | --- | --- | --- | --- | --- | --- | --- | --- | --- |
| 扁豆 | hyacinth bean | 蔬菜 | vegetables | 3.73 | 1.41 | 扁豆 | hyacinth bean | 豆角 | beans | 4.23 | 0.43 | 3.92 | 0.06 |
| 冬瓜 | wax gourd | 蔬菜 | vegetables | 4.13 | 1.17 | 冬瓜 | wax gourd | 西葫芦 | zucchini | 3.50 | 1.11 | 3.92 | 0.06 |
| 枸杞 | Chinese wolfberry | 蔬菜 | vegetables | 2.03 | 1.00 | 枸杞 | Chinese wolfberry | 山药 | yam | 2.57 | 1.33 | 3.78 | 0.06 |
| 白菜 | Chinese cabbage | 蔬菜 | vegetables | 4.57 | 0.50 | 白菜 | Chinese cabbage | 娃娃菜 | baby cabbage | 4.10 | 1.09 | 3.83 | 0.06 |
| 银耳 | tremella | 蔬菜 | vegetables | 2.83 | 1.26 | 银耳 | tremella | 枸杞 | Chinese wolfberry | 2.77 | 1.36 | 0.04 | 0.85 |
| 木瓜 | papaya | 蔬菜 | vegetables | 2.77 | 1.25 | 木瓜 | papaya | 南瓜 | pumpkin | 3.10 | 1.09 | 1.07 | 0.31 |
| 毛豆 | edamame | 蔬菜 | vegetables | 3.67 | 1.27 | 毛豆 | edamame | 豌豆 | peas | 4.03 | 0.61 | 1.98 | 0.17 |
| 青菜 | green vegetables | 蔬菜 | vegetables | 4.53 | 0.51 | 青菜 | green vegetables | 菠菜 | spinach | 4.33 | 0.48 | 3.96 | 0.10 |
| 金针菇 | needle mushroom | 蔬菜 | vegetables | 3.17 | 1.37 | 金针菇 | needle mushroom | 豆芽 | bean sprouts | 2.90 | 1.30 | 0.94 | 0.34 |
| 蒜苗 | garlic sprout | 蔬菜 | vegetables | 3.97 | 1.13 | 蒜苗 | garlic sprout | 蒜薹 | young garlic shoot | 4.00 | 0.69 | 0.02 | 0.89 |
| 紫菜 | nori | 蔬菜 | vegetables | 3.77 | 1.07 | 紫菜 | nori | 海带 | kelp | 3.60 | 1.22 | 0.32 | 0.58 |
| 豌豆 | peas | 蔬菜 | vegetables | 3.93 | 0.69 | 豌豆 | peas | 扁豆 | lentils | 3.90 | 1.16 | 0.39 | 0.85 |
| 蘑菇 | mushroom | 蔬菜 | vegetables | 3.53 | 1.31 | 蘑菇 | mushroom | 金针菇 | needle mushroom | 3.70 | 1.15 | 0.32 | 0.58 |
| 豆角 | beans | 蔬菜 | vegetables | 4.07 | 1.20 | 豆角 | beans | 毛豆 | edamame | 3.83 | 0.99 | 0.66 | 0.42 |
| 香椿 | cedar | 蔬菜 | vegetables | 4.07 | 0.94 | 香椿 | cedar | 芹菜 | celery | 3.93 | 0.83 | 0.41 | 0.53 |
| 姜 | ginger | 蔬菜 | vegetables | 3.10 | 1.42 | 姜 | ginger | 蒜 | garlic | 3.43 | 1.33 | 1.09 | 0.31 |
| 芦笋 | asparagus | 蔬菜 | vegetables | 4.07 | 0.94 | 芦笋 | asparagus | 竹笋 | bamboo shoot | 3.87 | 1.01 | 0.85 | 0.36 |
| 芋头 | taro | 蔬菜 | vegetables | 3.77 | 1.28 | 芋头 | taro | 红薯 | sweet potato | 4.03 | 0.85 | 1.00 | 0.33 |
| 螳螂 | mantis | 昆虫 | insects | 4.10 | 1.06 | 螳螂 | mantis | 蚱蜢 | grasshopper | 3.87 | 1.17 | 0.77 | 0.39 |
| 屎壳郎 | dung beetle | 昆虫 | insects | 4.10 | 1.03 | 屎壳郎 | dung beetle | 甲虫 | beetle | 4.00 | 0.91 | 0.28 | 0.60 |
| 蝗虫 | locust | 昆虫 | insects | 4.23 | 0.77 | 蝗虫 | locust | 蟋蟀 | cricket | 3.87 | 0.90 | 2.72 | 0.11 |
| 蟋蟀 | cricket | 昆虫 | insects | 4.33 | 0.71 | 蟋蟀 | cricket | 螳螂 | mantis | 4.00 | 0.98 | 3.63 | 0.07 |
| 蚱蜢 | grasshopper | 昆虫 | insects | 4.30 | 0.79 | 蚱蜢 | grasshopper | 蝗虫 | locust | 3.93 | 1.01 | 2.72 | 0.11 |
| 苍蝇 | fly | 昆虫 | insects | 3.90 | 1.16 | 苍蝇 | fly | 蚊子 | mosquito | 3.70 | 1.18 | 0.48 | 0.50 |
| 甲虫 | beetle | 昆虫 | insects | 4.47 | 0.51 | 甲虫 | beetle | 瓢虫 | beetle | 4.20 | 0.61 | 3.46 | 0.07 |
| 山楂 | hawthorn | 水果 | fruit | 3.53 | 1.28 | 山楂 | hawthorn | 枣 | jujube | 3.23 | 1.14 | 1.08 | 0.31 |
| 黄瓜 | cucumber | 水果 | fruit | 2.97 | 1.38 | 黄瓜 | cucumber | 西红柿 | tomato | 2.67 | 1.30 | 0.87 | 0.36 |
| 橄榄 | olive | 水果 | fruit | 3.43 | 1.33 | 橄榄 | olive | 柠檬 | lemon | 3.07 | 1.14 | 1.75 | 0.20 |
| 柚子 | grapefruit | 水果 | fruit | 4.47 | 0.68 | 柚子 | grapefruit | 橙子 | orange | 4.17 | 0.79 | 2.42 | 0.13 |
| 核桃 | walnut | 水果 | fruit | 2.60 | 1.40 | 核桃 | walnut | 栗子 | chestnut | 3.07 | 1.14 | 3.68 | 0.07 |
| 枣 | jujube | 水果 | fruit | 3.23 | 1.38 | 枣 | jujube | 圣女果 | cherry tomato | 3.07 | 1.14 | 0.40 | 0.53 |
| 仓鼠 | hamster | 哺乳类 | mammals | 3.97 | 1.10 | 仓鼠 | hamster | 老鼠 | mouse | 4.1 | 0.84 | 0.41 | 0.53 |
| 海豚 | dolphin | 哺乳类 | mammals | 3.93 | 1.28 | 海豚 | dolphin | 鲸鱼 | whale | 3.7 | 0.90 | 0.53 | 0.47 |
| 老虎 | tiger | 哺乳类 | mammals | 4.43 | 0.73 | 老虎 | tiger | 狮子 | lion | 4.07 | 0.98 | 3.78 | 0.06 |
| 喜鹊 | magpie | 鸟类 | birds | 4.43 | 0.68 | 喜鹊 | magpie | 杜鹃 | cuckoo | 4.23 | 0.68 | 2.07 | 0.16 |
| 鹅 | goose | 鸟类 | birds | 3.37 | 1.35 | 鹅 | goose | 天鹅 | swan | 3.80 | 1.16 | 2.36 | 0.14 |
| 蜂鸟 | hummingbird | 鸟类 | birds | 4.03 | 1.00 | 蜂鸟 | hummingbird | 画眉 | thrush | 3.67 | 0.99 | 2.86 | 0.10 |
| 杜鹃 | cuckoo | 鸟类 | birds | 4.50 | 0.68 | 杜鹃 | cuckoo | 喜鹊 | magpie | 4.23 | 0.68 | 2.07 | 0.16 |
| 鸡 | chick | 鸟类 | birds | 3.13 | 1.41 | 鸡 | chicken | 鸭 | duck | 3.90 | 0.96 | 7.37 | 0.11 |
| 画眉 | thrush | 鸟类 | birds | 4.00 | 0.72 | 画眉 | thrush | 翠鸟 | kingfisher | 4.17 | 0.75 | 2.73 | 0.11 |
| 扁豆 | hyacinth bean | 服装 | clothing | 1.37 | 0.49 | 扁豆 | hyacinth bean | 披肩 | tippet | 1.33 | 0.48 | 0.11 | 0.75 |
| 冬瓜 | wax gourd | 服装 | clothing | 1.27 | 0.45 | 冬瓜 | wax gourd | 军装 | military uniform | 1.20 | 0.41 | 0.66 | 0.42 |
| 枸杞 | Chinese wolfberry | 服装 | clothing | 1.33 | 0.71 | 枸杞 | Chinese wolfberry | 棉裤 | cotton-padded trousers | 1.43 | 0.50 | 0.59 | 0.45 |
| 白菜 | Chinese cabbage | 服装 | clothing | 1.40 | 0.81 | 白菜 | Chinese cabbage | 棉袄 | cotton-padded jacket | 1.63 | 0.85 | 0.85 | 0.21 |
| 银耳 | tremella | 服装 | clothing | 1.43 | 0.77 | 银耳 | tremella | 秋衣 | long underwear | 1.77 | 1.07 | 2.50 | 0.13 |
| 木瓜 | papaya | 服装 | clothing | 1.33 | 0.66 | 木瓜 | papaya | 婚纱 | bridal gown | 1.33 | 0.71 | 0.00 | 0.99 |
| 毛豆 | edamame | 服装 | clothing | 1.40 | 0.86 | 毛豆 | edamame | 开衫 | cardigan | 1.43 | 0.86 | 0.51 | 0.82 |
| 青菜 | green vegetables | 服装 | clothing | 1.50 | 0.94 | 青菜 | green vegetables | 礼服 | full dress | 1.70 | 1.02 | 1.13 | 0.30 |
| 金针菇 | needle mushroom | 服装 | clothing | 1.33 | 0.48 | 金针菇 | needle mushroom | 连体裤 | jumpsuits | 1.37 | 0.49 | 0.11 | 0.75 |
| 蒜苗 | garlic sprout | 服装 | clothing | 1.23 | 0.63 | 蒜苗 | garlic sprout | 短裙 | miniskirt | 1.33 | 0.66 | 0.38 | 0.54 |
| 紫菜 | nori | 服装 | clothing | 1.27 | 0.52 | 紫菜 | nori | 卫衣 | hoodie | 1.33 | 0.66 | 1.00 | 0.33 |
| 豌豆 | peas | 服装 | clothing | 1.80 | 1.03 | 豌豆 | peas | 短裤 | shorts | 1.90 | 0.99 | 0.24 | 0.63 |
| 蘑菇 | mushroom | 服装 | clothing | 1.57 | 0.90 | 蘑菇 | mushroom | 毛衣 | sweater | 1.47 | 0.78 | 0.59 | 0.45 |
| 豆角 | beans | 服装 | clothing | 1.43 | 0.94 | 豆角 | beans | 旗袍 | Chinese dress | 1.50 | 0.73 | 0.11 | 0.74 |
| 香椿 | cedar | 服装 | clothing | 1.80 | 1.13 | 香椿 | cedar | 衬衫 | shirt | 2.00 | 1.26 | 1.00 | 0.33 |
| 姜 | ginger | 服装 | clothing | 1.17 | 0.38 | 姜 | ginger | 长裤 | trousers | 1.30 | 0.53 | 2.83 | 0.10 |
| 芦笋 | asparagus | 服装 | clothing | 2.07 | 1.20 | 芦笋 | asparagus | 短袖 | short sleeve | 1.77 | 0.86 | 2.58 | 0.12 |
| 芋头 | taro | 服装 | clothing | 1.30 | 0.79 | 芋头 | taro | 大衣 | overcoat | 1.13 | 0.35 | 1.50 | 0.23 |
| 螳螂 | mantis | 电器 | electric appliance | 1.47 | 0.94 | 螳螂 | mantis | 风扇 | fan | 1.27 | 0.52 | 1.85 | 0.18 |
| 屎壳郎 | dung beetle | 电器 | electric appliance | 1.27 | 0.78 | 屎壳郎 | dung beetle | 洗衣机 | washing machine | 1.27 | 0.52 | 0.00 | 0.99 |
| 蝗虫 | locust | 电器 | electric appliance | 1.20 | 0.41 | 蝗虫 | locust | 电脑 | computer | 1.20 | 0.61 | 0.00 | 0.99 |
| 蟋蟀 | cricket | 电器 | electric appliance | 1.33 | 0.80 | 蟋蟀 | cricket | 电视 | TV | 1.57 | 1.07 | 2.45 | 0.13 |
| 蚱蜢 | grasshopper | 电器 | electric appliance | 1.33 | 0.66 | 蚱蜢 | grasshopper | 冰箱 | refrigerator | 1.40 | 0.93 | 0.33 | 0.57 |
| 苍蝇 | fly | 电器 | electric appliance | 1.70 | 0.88 | 苍蝇 | fly | 空调 | air conditioning | 1.50 | 0.86 | 3.22 | 0.08 |
| 甲虫 | beetle | 电器 | electric appliance | 1.37 | 0.81 | 甲虫 | beetle | 烤箱 | oven | 1.30 | 0.65 | 0.24 | 0.63 |
| 山楂 | hawthorn | 兵器 | weapons | 1.87 | 1.01 | 山楂 | hawthorn | 长枪 | pike | 1.73 | 0.74 | 0.89 | 0.35 |
| 黄瓜 | cucumber | 兵器 | weapons | 1.90 | 1.18 | 黄瓜 | cucumber | 剑 | sword | 1.80 | 0.96 | 0.59 | 0.45 |
| 橄榄 | olive | 兵器 | weapons | 1.57 | 0.77 | 橄榄 | olive | 冲锋枪 | submachine gun | 1.53 | 0.86 | 0.08 | 0.79 |
| 柚子 | grapefruit | 兵器 | weapons | 1.53 | 0.86 | 柚子 | grapefruit | 狙击枪 | sniper rifle | 1.50 | 0.86 | 0.09 | 0.77 |
| 核桃 | walnut | 兵器 | weapons | 1.43 | 0.82 | 核桃 | walnut | 步枪 | rifle | 1.57 | 1.04 | 0.46 | 0.50 |
| 枣 | jujube | 兵器 | weapons | 1.77 | 1.10 | 枣 | jujube | 手枪 | pistol | 1.67 | 0.99 | 0.35 | 0.56 |
| 仓鼠 | hamster | 家具 | furniture | 1.77 | 1.04 | 仓鼠 | hamster | 衣柜 | wardrobe | 1.53 | 0.86 | 2.03 | 0.17 |
| 海豚 | dolphin | 家具 | furniture | 2.20 | 1.27 | 海豚 | dolphin | 沙发 | sofa | 1.90 | 0.99 | 2.16 | 0.15 |
| 老虎 | tiger | 家具 | furniture | 2.00 | 1.26 | 老虎 | tiger | 床 | bed | 1.60 | 0.81 | 3.08 | 0.09 |
| 喜鹊 | magpie | 工具 | tool | 1.67 | 1.03 | 喜鹊 | magpie | 铁锹 | spade | 1.77 | 1.14 | 0.42 | 0.52 |
| 鹅 | goose | 工具 | tool | 1.47 | 0.94 | 鹅 | goose | 锄头 | hoe | 1.23 | 0.43 | 1.34 | 0.26 |
| 蜂鸟 | hummingbird | 工具 | tool | 1.60 | 0.86 | 蜂鸟 | hummingbird | 铲子 | shovel | 1.57 | 0.82 | 0.03 | 0.86 |
| 杜鹃 | cuckoo | 工具 | tool | 1.27 | 0.64 | 杜鹃 | cuckoo | 钳子 | pliers | 1.3 | 0.60 | 0.04 | 0.84 |
| 鸡 | chick | 工具 | tool | 1.23 | 0.43 | 鸡 | chick | 锤子 | hammer | 1.27 | 0.52 | 0.11 | 0.75 |
| 画眉 | thrush | 工具 | tool | 1.27 | 0.58 | 画眉 | thrush | 扳手 | spanner | 1.37 | 0.81 | 0.35 | 0.56 |
